# Impact of the stringency of lockdown measures on covid-19: A theoretical model of a pandemic

**DOI:** 10.1371/journal.pone.0258205

**Published:** 2021-10-05

**Authors:** Claudio Violato, Emilio Mauro Violato, Efrem Mauro Violato

**Affiliations:** 1 Professor and Assistant Dean, University of Minnesota Medical School, Minneapolis, MN, United States of America; 2 Department of Psychology, KU Leuven University, Leuven, Belgium; 3 Department of Educational Psychology, University of Alberta, Edmonton, AB, Canada; University "Magna Graecia" of Catanzaro, ITALY

## Abstract

**Background:**

How effective have lockdowns been at reducing the covid-19 infection and mortality rates? Lockdowns influence contact among persons within or between populations including restricting travel, closing schools, prohibiting public gatherings, requiring workplace closures, all designed to slow the contagion of the virus. The purpose of the present study was to assess the impact of lockdown measures on the spread of covid-19 and test a theoretical model of the covid-19 pandemic employing structural equation modelling.

**Methods:**

Lockdown variables, population demographics, mortality rates, infection rates, and health were obtained for eight countries: Austria, Belgium, France, Germany, Italy, Netherlands, Spain, and the United Kingdom. The dataset, owid-covid-data.csv, was downloaded on 06/01/2020 from: https://github.com/owid/covid-19-data/tree/master/public/data. Infection spread and mortality data were depicted as logistic growth and analyzed with stepwise multiple regression. The overall structure of the covid-19 data was explored through factor analyses leading to a theoretical model that was tested using latent variable path analysis.

**Results:**

Multiple regression indicated that the time from lockdown had a small but significant effect (β = 0.112, p< 0.01) on reducing the number of cases per million. The stringency index produced the most important effect for mortality and infection rates (β = 0.588,β = 0.702, β = 0.518, β = 0.681; p< 0.01). Exploratory and confirmatory analyses resulted in meaningful and cohesive latent variables: 1) *Mortality*, 2) *Infection Spread*, 3) *Pop Health Risk*, and 4) *Health Vulnerability* (Comparative Fit Index = 0.91; Standardized Root Mean Square Residual = 0.08).

**Discussion:**

The stringency index had a large impact on the growth of covid-19 infection and mortality rates as did percentage of population aged over 65, median age, per capita GDP, diabetes prevalence, cardiovascular death rates, and ICU hospital beds per 100K. The overall Latent Variable Path Analysis is theoretically meaningful and coherent with acceptable fit indices as a model of the covid-19 pandemic.

## Introduction

How effective have lockdowns been at reducing the covid-19 infection and mortality rates? Now that many countries have had more than a year under lockdown, it is possible to study empirically the effectiveness of lockdowns in reducing the spread of covid-19 and the related mortality [1–5]. These lockdowns are non-pharmaceutical interventions of contact among persons within or between populations including restricting travel, closing schools, prohibiting public gatherings, requiring workplace closures, all designed to slow the contagion of the virus. In a recent (published June 2021) large-scale study employing a spatiotemporal analysis of human mobility during the COVID-19 epidemic in Switzerland, it was found that a 1% reduction in human mobility predicted a 0.88 to 1.11% reduction in daily reported COVID-19 cases [1]. But even as the pandemic has abated in some countries with slowing infection and mortality, it has sped up in some African countries, Columbia, and Brazil [1, 2]. The coronavirus appeared in China and then spread to Europe, first in Italy then Spain and subsequently to other countries [6].

Most of anti-contagion approaches are primarily informed by epidemiological simulations [7–9] that can be useful in modeling the impact of policies such as lockdowns, but the actual effects of these policies on infection rates in the ongoing covid-19 pandemic are not well understood. Some empirical studies of the spread and rate of infection of the covid-19 have been done.

An early study of the pandemic by Tobías [10] found that from the end of February through April 2020, the SARS-CoV-2 epidemic in Spain followed the pattern of that in Italy very closely. Quasi-Poisson regression analyses showed that after the first lockdown, incidence trends were reduced in both countries for some outcomes (e.g., new cases, total deaths, deaths per million, etc.) but the infection trends kept rising. During a second lockdown the trend slopes for both countries in daily incident cases and intensive care unit admissions were reduced when more restrictive measures for mobility were introduced. The *stringency* of lockdown may be effective in slowing the spread. In simulation models using piecewise functions, results indicated that lockdowns could reduce the spread of covid-19 infection [11].

Ambikapathy and Krishnamurthy [12] assessed the validity of the impact of various lockdown scenarios (14, 21, 42 days) on covid-19 transmission in India. For high interpersonal contact (e.g., crowded transit stations) resulting in exposure to infection, the model predicted an exponential transmission. Hsiang et al [6] employed panel regression models to estimate how the daily growth rate of infections changes over time within China, South Korea, Italy, Iran, France, and the United States (US). Interventions such as travel restrictions, social distancing, quarantines, lockdowns, and closing schools prevented or delayed several million cases.

There are several major factors such as timeliness, duration and stringency of lockdown that can affect the spread and mortality of covid-19 infection. The stringency index (Government Response Stringency Index) is a composite measure based on 9 response indicators including school closures, workplace closures, and travel bans, rescaled to a value from 0 to 100 (100 = strictest response—Oxford COVID-19 Government Response Tracker, Blavatnik School of Government) (see S1 Appendix for a full description).

The impact of a full lockdown with very rigorous stringency was demonstrated in Vo’, a small town of around 3,000 people near Venice in the Veneto region [13]. In response to the first confirmed covid-19 death in Italy on February 21, 2020, in Vo’ the regional authorities imposed a lockdown of the whole municipality for 14 days. Lavezzo et al [13] collected information on the demographics, clinical presentation, hospitalization, contact network and presence of SARS-CoV-2 infection in nasopharyngeal swabs for the population at two consecutive time points. The results indicated that the testing and tracing approach had a huge impact on the course of the epidemic essentially stopping the transmission of the virus.

The findings from the above empirical studies and simulations may help inform policies and when they should be deployed, intensified, or lifted. None of this work, however, has provided an urgently needed systematic theoretical model of how the covid-19 pandemic is behaving. A structural equation modeling approach, using latent variable path analysis (LVPA), may provide such a model.

### Structural Equation Modelling (SEM): Latent variable path analysis

SEM represents major advance in data analysis and theory building and testing in social and psychological data, but has not been widely used by epidemiologists. LVPA, a specific application of SEM, is a method to identify and assess the effects of a set of variables acting on a specified outcome via multiple causal pathways. With this powerful statistical tool, complex relationships among observed and latent variables can be analyzed. Additionally, causal relationships with non-experimental data can be posited and tested. This allows researchers to explain the development of phenomena such as disease and health behaviors. These statistical methods include factor analysis and structural equation models. SEM is a significant advance over conventional epidemiological analytic techniques such as multiple regression, path analyses, exploratory factor analyses, etc., as it examines linear causal relationships among variables, while simultaneously accounting for measurement error. Therefore, LVPA was applied to the covid-19 epidemiological data to develop and test a theoretical model of the pandemic.

The major purposes of the present study were to (1) explore the timeliness and severity of lockdown in eight European countries (Austria, Belgium, France, Germany, Italy, Netherlands, Spain and United Kingdom) to see how effective the lockdown has been in reducing the spread of covid-19 and the related mortality; and (2) to employ advanced epidemiological research methods by exploring the structure of the data to develop and test the fit of a model of the covid-19 pandemic through LVPA.

## Method

### Data

The dataset, owid-covid-data.csv, was downloaded on 06/01/2020 from: https://github.com/owid/covid-19-data/tree/master/public/data (The complete COVID-19 dataset is a collection of the COVID-19 data maintained by *Our World in Data—*Statistics and Research and data: Hannah Ritchie, Esteban Ortiz-Ospina, Diana Beltekian, Edouard Mathieu, Joe Hasell, Bobbie Macdonald, Charlie Giattino, and Max Roser; Web development: BreckYunits, Ernst van Woerden, Daniel Gavrilov, MatthieuBergel, Shahid Ahmad, and Jason Crawford). The variables are summarized in Table 1 (details for data columns and variables are in S2 Appendix).

**Table 1:** Descriptive statistics for each country through to June 1, 2020.

Variable	Austria	Belgium	France	Germany	Italy	Netherlands	Spain	UK
Total Cases	16642	58381	151753	181815	233019	46442	239429	274762
Total cases / mil	780.62	1673.45	792.78	743.39	1591.85	960.93	1910.65	1063.84
New cases / mil	17.61	24.02	18.36	30.88	29.05	17.61	24.02	18.36
Total deaths	668	9467	28802	8511	33415	5956	27127	38489
Total deaths/ mil	74	251.27	136.62	26.13	214.11	115.01	204.88	154.33
New deaths / mil	.40	4.72	3.28	.70	3.62	2.22	3.81	4.15
Stringency index	39.77	44.04	49.15	38.29	56.10	38.90	46.22	37.13
Median age	44.40	41.80	42.00	46.60	47.90	43.20	45.50	40.80
% aged 65 or older	19.20	18.57	19.72	21.45	23.02	18.78	19.44	18.52
CV death rate	145.18	114.90	86.06	156.14	113.15	109.36	99.40	122.14
ICU hospital beds/100k	7.37	5.64	5.98	8.00	3.18	3.32	2.97	2.54
Days since lockdown	76	74	75	69	83	69	78	68
gdp per capita	45436	42658	38605	45229	35220	48472	34272	39753
Diabetes prevalence	6.25	4.29	4.77	8.31	4.78	5.29	7.17	4.28

### Analyses

The total sample from December 31, 2019 to June 1, 2020 consisted of 1,231 cases. This sample was randomly separated into two sub-samples. This dual sampling procedure has been recommended when employing structural equation modeling to allow both exploratory and confirmatory techniques [14, 15]. The first random sample (n = 616) was used to conduct hierarchical logistic analyses and stepwise multiple regression (SMR) analyses. Additionally, exploratory factor analysis (EFA) with principal components as the extraction method and oblique (oblimin) rotations were run. SPSS 24.0 was used for these analyses employing two-tailed tests with p < .05.

A latent variable path analysis (LVPA) was conducted on the second random sample (n = 615). The structural equation computer program *lavaan* from Comprehensive R Archive Network (CRAN - http://CRAN.R-project.org/package=lavaan and supported by the website http://lavaan.org/.) was used for the LVPA as it is a specialized and more flexible program for these type of analyses than is SPSS. Several fit indices were selected in order to test the model: comparative fit index (CFI), Tucker Lewis Index (TLI), and standardized root mean square residual (SRMR) [14, 15]. Typically, good fitting models have an SRMR at or less than 0.08, CFI greater than 0.90 and TLI greater than 0.85 [16].

In summary, three types of analyses were conducted to (1) assess the influence of the lockdown measures (hierarchical logistic models) of the covid-19 infection of the mortality data for eight countries (Austria, Belgium, France, Germany, Italy, Netherlands, Spain, and the United Kingdom), (2) determine the best predictors of mortality and infection spread (SMR), and (3) explore (EFA) and test the fit of a theoretically comprehensive model of the latent structure of the covid-19 data (LVPA).

## Results

### Descriptive statistics and logistic models of the mortality data for the eight countries

Table 1 contains summary data for the 8 countries. The highest total cases was in Spain (239,429) with Italy closely behind (233,019). The fewest total case was in Austria (16,642). When comparing the prevalence rates the highest rate of cases / million was Spain (1,910.65) with Belgium (1,673.45) a close second; Germany had the lowest prevalence (743.39). The stringency index is the composite measure. A number of other variables (median age of the population, % aged 65 or older, cardiovascular death rate, diabetes prevalence, etc.) are summarized in Table 1.

**Fig 1:**
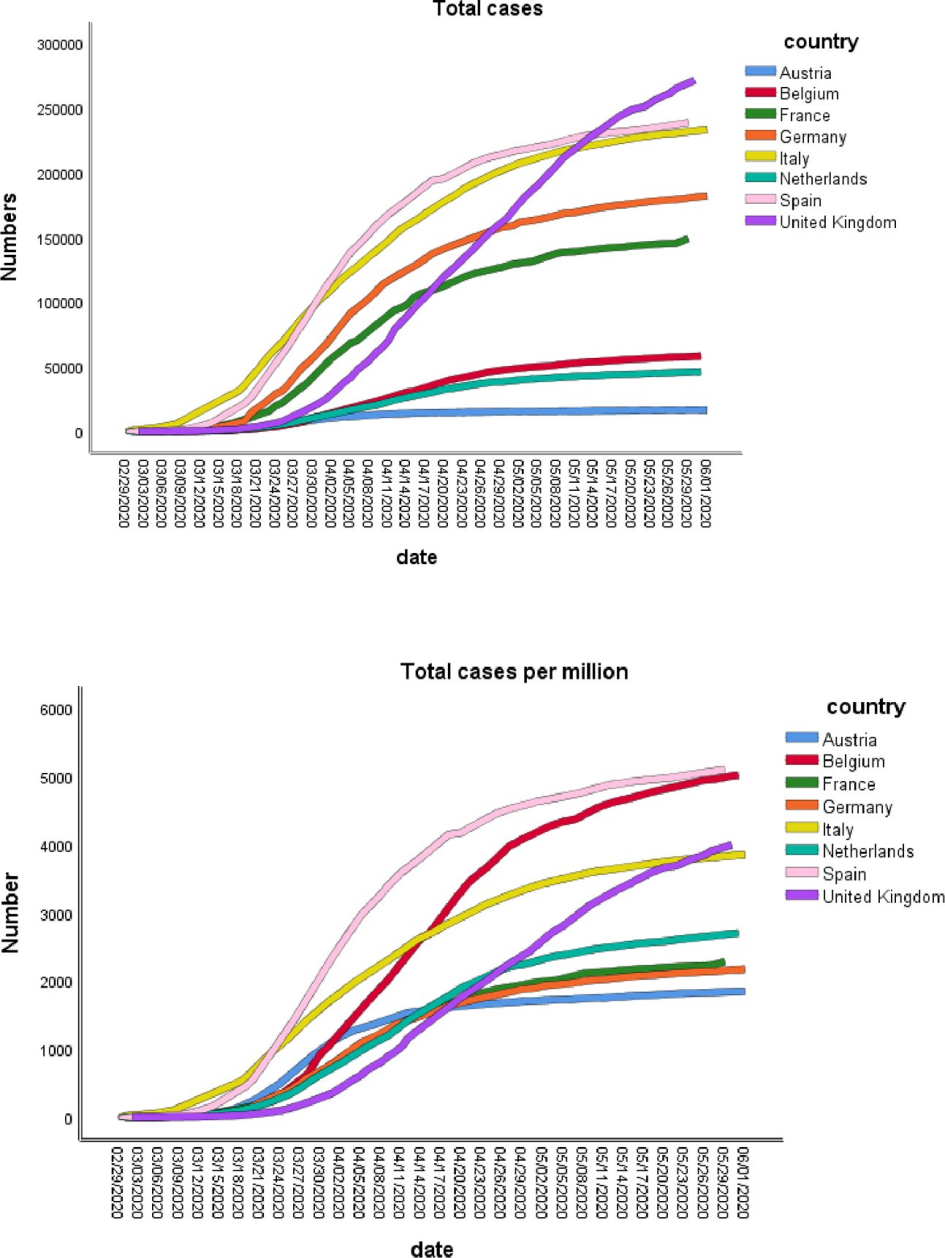
Cases over time. Lockdown dates: Austria = 2020-03-16, Belgium = 2020-03-18, France = 2020-03-17, Germany = 2020-03-23, Italy = 2020-03-09, Netherlands = 2020-03-23, Spain = 2020-03-14, United Kingdom = 2020-03-24.

Fig 1 contains the logistic growth data for the eight countries from February 29 to June 1, 2020. Additionally, the lockdown dates are summarized in the figure. The supposition is that early lockdown helps to “flatten the curve” or reduce the rate of deaths per million. The earliest lockdown was Italy (March 9) and the latest was the United Kingdom (March 24).

The mean incubation period (the time from exposure to symptom onset) for COVID-19 is approximately 5 (2–7) days [17, 18]. Approximately 98% of individuals who develop symptoms will do so within 11.5 days of infection. The median interval from symptom onset to hospital admission is 7 (3–9) days. The onset of symptoms that result in death is 6 to 41 days with a median = 14 days [18]. Therefore, in Fig 1 when the first deaths from covid-19 were detected in these European countries in late February and early March, the infection was well underway. The first cases were identified in early March in Italy and mid-March in Spain (Fig 1). Logistic growth of cases continued in both of these countries until mid-April and began to slow after that.

### Prevalence

**Fig 2:**
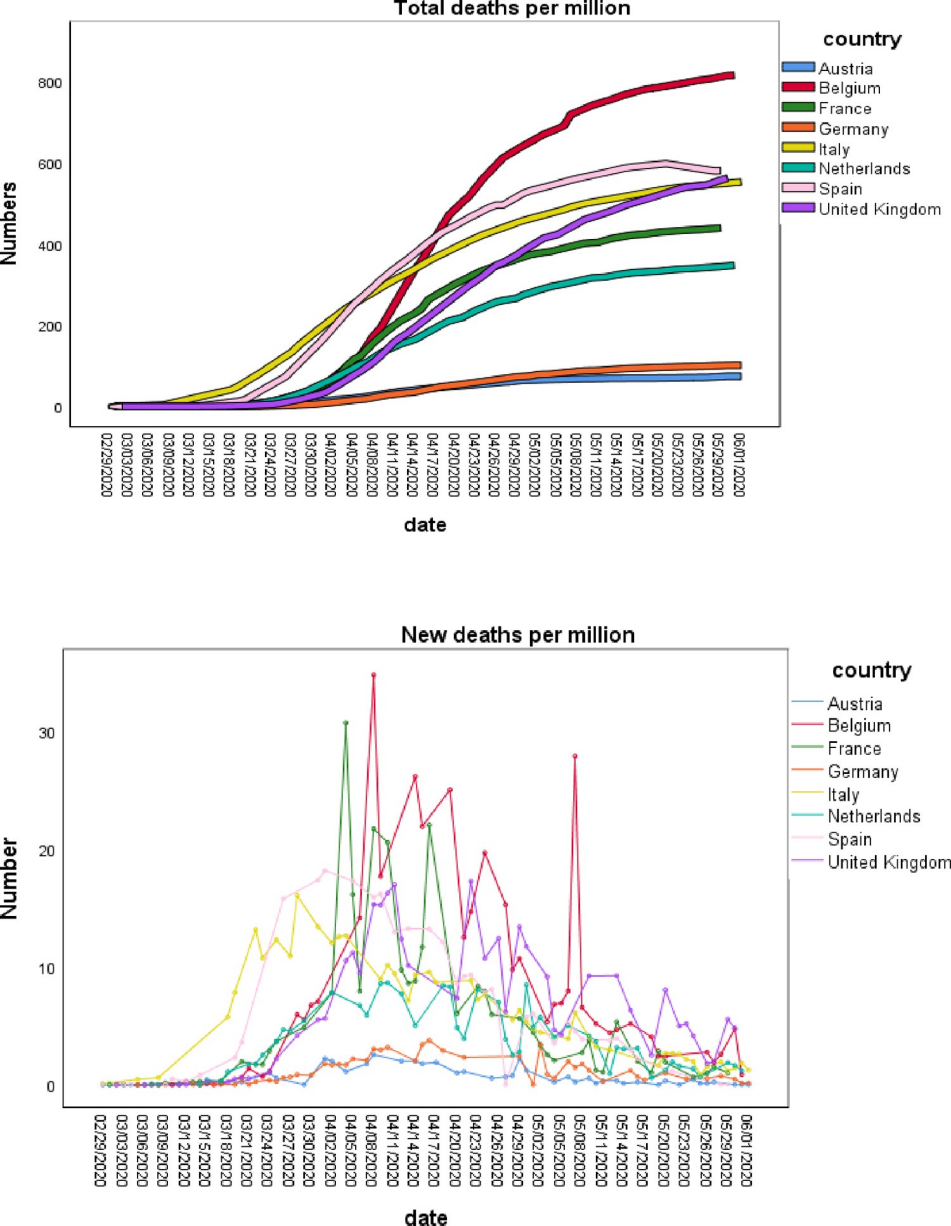
Deaths over time.

Prevalence is the number of cases of covid-19 that are present in a particular population (e.g., country) at a given time. The death rates per million over time are summarized in Fig 2. France’s deaths began around mid-March but the rate was slower than for Italy and Spain. France’s curve started to flatten in mid-May. The total growth of number of cases was slower for Belgium, Netherlands, Germany and Austria (except for Germany, all relatively small populations, Belgium = 11.5 million; Netherlands = 17 million; Austria = 9 million). In the United Kingdom (population = 68 million) the number of deaths did not start to grow rapidly until early April but then increased sharply and continued to June.

### Incidence

Incidence is the number of new deaths of covid-19 that develop in a given period of time, in Fig 2, the new deaths per million. The incidence rates are highly variable by country with Spain and Italy showing high incidence in the earlier part of the year, France later and the remaining countries later still.

### Flattening the curve

Given the incubation period to hospitalization of roughly 14 days of Covid-19 and 14 days beyond that for death to occur, a “flattening of the curve” should be observed within about 28 days to one month of lockdown. In Fig 1 (Total cases), we do see such a phenomenon for Austria and Germany but not for Italy, France, Spain and Belgium. The Netherlands appears to be intermediate in this. The total deaths per million (Fig 1) follow a logistic growth for Italy, France, the UK, and Spain but flatter curves for Austria, Germany, Netherlands and Belgium. The rate of new deaths per million is highly variable across countries after lockdown. Lockdown timing does not seem to have a straightforward effect of “flattening the curve” or slowing the mortality rate.

Belgium is an anomaly when comparing Fig 1 (Total cases per million) and Fig 2 (Total deaths per million), the growth curves for cases and deaths are rapid (Figs 1 and 2). The infection rate resulting in deaths per capita in Belgium appears unusually high compared to the other European countries.

A summary of the above observations indicates that early lockdown has only a minor effect on the rate of growth of deaths per million and total cases per million. A possible major factor to be explored for reducing the rate of growth for deaths is the stringency index. Both the UK and Germany had among the lowest stringency indices (37.13 and 38.29 respectively—Table 1) perhaps accounting for the rapid growth of deaths per million and new deaths per million for the UK but not for Germany.

### Stepwise multiple regression analyses

In order to determine the impact of several variables simultaneously on mortality data and infection rate, four SMR analyses were conducted for three important mortality variables (total deaths per million, total cases per million, and total deaths) and the infection rate (new cases per million)—Table 2. The stepwise criteria were Probability-of-F-to-enter ≤ 0.05, Probability-of-F-to-remove ≥ 0.10 for all the beginning independent variables: stringency index, ICU hospital beds per100k, gdp per capita, diabetes prevalence, days since lockdown, aged 65 or older, median age, and cvd death rate.

**Table 2:** Stepwise* multiple regression for covid-19 cases and deaths.

**Part A–Dependent variable: total deaths per million**			
Model: Independent variables	R^£^	R^2^	β^€^
1. stringency index	.609	.370	.588^a^
2. stringency index, ICU hospital beds per100k	.646	.418	-.107
3. stringency index, ICU hospital beds per100k, diabetes prevalence	.654	.427	-.119
4. stringency index, ICU hospital beds per100k, diabetes prevalence, gdp per capita	.661	.437	-.112
**Part B–Dependent variable: total cases per million**			
Model: Independent variables	R^£^	R^2^	β^€^
1. stringency index	.707	.500	.702 ^a^
2. stringency index, ICU hospital beds per100k	.721	.520	-.124
3. stringency index, ICU hospital beds per100k, days since lockdown	.726	.528	.112
4. stringency index, ICU hospital beds per100k, days since lockdown, aged 65 or older	.730	.532	-.243
5. stringency index, ICU hospital beds per100k, days since lockdown, aged 65 or older, median age	.736	.542	-.203
**Part C–Dependent variable: total deaths**			
Model: Independent variables	R^£^	R^2^	β^€^
1. stringency index	.558	.312	.518 ^a^
2. stringency index, gdp per capita	.664	.441	-.239
3. stringency index, gdp per capita, ICU hospital beds per100k	.676	.457	-.172
4. stringency index, gdp per capita, ICU hospital beds per100k, aged 65 older	.679	.461	.215
**Part D–Dependent variable: new cases per million**			
Model: Independent variables	R^£^	R^2^	β^€^
1. stringency index	.673	.453	.681 ^a^
2. stringency index, ICU hospital beds per100k	.683	.467	-.144
3. stringency index, ICU hospital beds per100k, cvd death rate	.687	.472	.161
4. stringency index, ICU hospital beds per100k, cvd death rate, aged 65 or older	.691	.478	-.137
5. stringency index, ICU hospital beds per100k, cvd death rate, aged 65 or older, gdp per capita	.696	.484	-.117

*Stepwise Criteria: Probability-of-F-to-enter ≤ 0.05, Probability-of-F-to-remove ≥ 0.10).

^£^ Multiple correlation coefficient

^€^ Standardized beta weight (ranges: +1.0 to -1.0)

^a^Beta for Stringency Index.

Part A, B and C of Table 2 contain prevalence information. The stepwise regression models in Part A has total deaths per million as the dependent variable. The optimal results is model 4 with stringency index (β = 0.588, p< 0.01), ICU hospital beds per100k (β = -0.107, p< 0.01), gdp per capita (β = -0.119, p< 0.01), diabetes prevalence (β = -0.112, p< 0.01) with a Multiple R = 0.661 and R^2^ = 0.437 (i.e., 43.7% of the variance).

Table 2, Part B has total cases per million with 5 independent variables: stringency index (β = 0.702, p < .01), ICU hospital beds per100k (β = -0.124, p < .01), days since lockdown (β = 0.112, p < .01), aged 65 or older (β = 0.243, p < .01), and median age (β = -0.203, p < .01). This model results in a multiple R = 0.736 and R^2^ = 0.542 (54.1% of the variance). The regression analysis for total deaths (independent variable) is summarized in Part C of Table 2. The optimal model consists of stringency index (β = 0.518, p < .01), gdp per capita (β = -0.239, p < .01), ICU hospital beds per100k (β = -0.172, p< .01), and aged 65 or older (β = 0.215, p < .01). This model results in a Multiple R = 0.679 and R^2^ = 0.461 (i.e., 46.1% of the variance).

Incidence (Part D of Table 2) contains the dependent variable, new cases per million, and five independent variables resulting in multiple R = 0.696 and R^2^ = 0.484 (48.4% of the variance): stringency index (β = 0.681p < .01), ICU hospital beds per100k (β = - 0.144, p < .01), cardiovascular death rate [CVD] (β = 0.161, p < .01), aged 65 or older (β = -0.137, p < .01), gdp per capita (β = -0.117, p < .01) and known to have an impact on mortality rates for covid-19 infections [19, 20].

The stringency index is the single most important independent variable in all four of the SMRs (i.e., Parts A, B, C, D of Table 2). Days since lockdown also figures as a significant IV in the total cases per million (Part B) regression analysis. (The full regression equations for raw data and un-standardized beta weights that allow for predictions of outcomes are reported in S3 Appendix). The predicted results for total deaths per million, total cases per million, and total deaths are linear functions correlated with actual outcomes at r = .83, r = .78, and r = .83 respectively. For new cases per million, there is also a linear function resulting in r = .34 of predicted to actual outcomes. The predictive efficiency of these regression equations is summarized in S4 Appendix.

### Exploratory factor analysis and latent variable path analysis

#### Exploratory factor analysis

Exploratory factor analysis (EFA) was employed to study the structure of the covid-19 data, particularly demographic, social and epidemiological variables to explain correlations among multiple variables as underlying factors. Principal component analysis (PCA) was employed as the extraction method. Direct oblimin rotation with Kaiser Normalization was employed to achieve a simple factor structure and to explore the correlations among factors (see S5 Appendix for full matrix).

An optimal solution resulted in four cohesive, theoretically meaningful factors that accounted for 84.6% of the total variance: 1) *Infection Spread*, 2) *Health Vulnerability*, 3) *Pop Health Risk*, and 4) *Mortality*. These factors represent underlying concepts that cannot be adequately measured by a single variable. For example, various measures of *Infection Spread* may be influenced by one or more underlying factors (new cases, new deaths, new deaths per million, stringency index).

*Infection Spread* accounted for the largest percentage of the variance (35.8%). *Health Vulnerability* with loadings from median age, aged 65 years or older, and days since lockdown accounted for 21.6% of the variance. The major loadings on *Pop Health Risk* were cardiovascular death rate, ICU hospital beds per 100k, with minor contributions (split loading) from days since lockdown for 15.01% of the variance. *Mortality* had loadings from total deaths per million, total cases per million and a split loading from the stringency index, accounting for 12.3% of the variance. Both of the lockdown variables (days since lockdown; and how stringently society was locked down) loaded as theoretically expected. The stringency index loaded on both *Infection Spread* and *Mortality*, which were correlated r = .41 (p < .01). *Health Vulnerability* was correlated with *Pop Health Risk*, r = .14 (p < .05.

### Latent variable path analysis

**Fig 3:**
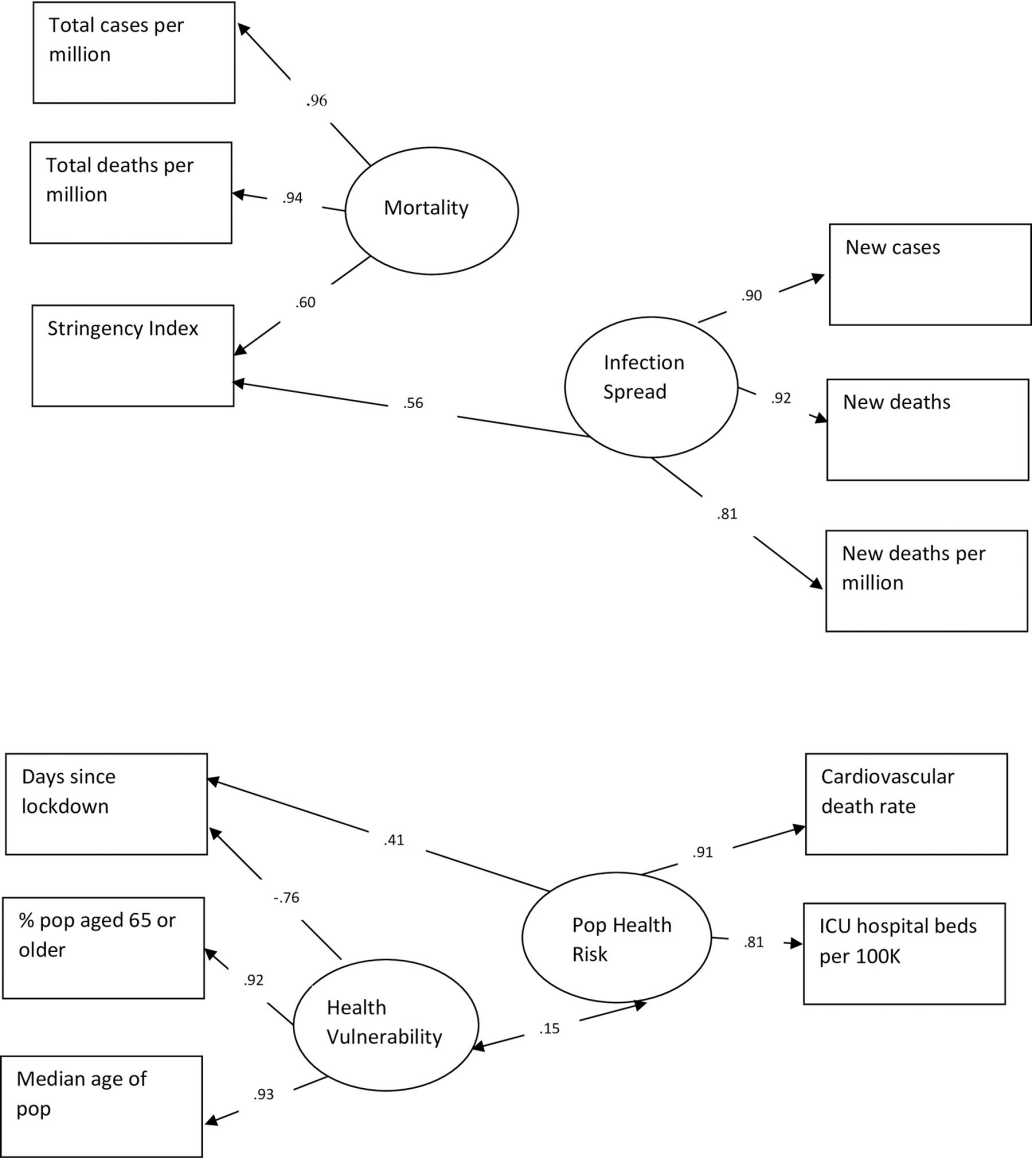
Latent variable path analysis of covid-19 impact using Maximum Likelihood (ML) estimation. (Comparative Fit Index = 0.91; Tucker-Lewis Index = 0.85; Standardized Root Mean Square Residual = 0.08.

The next stage of the analysis employed LVPA to investigate the construct validity of the covid-19 data structure. Based on the measurement model derived in the EFA, we developed the full latent variable model (Fig 3) and tested it on the second random sample of data (n = 615) with maximum likelihood (ML) estimation with the lavaan program in R (the R code and raw results for these analyses are summarized in S6 Appendix). The LVPA model showed an acceptable fit (CFI = 0.91; TLI = .85; SRMR = .08). Standardized path coefficients for the model are displayed in Fig 3. Four latent variables were identified and confirmed in the model: 1) *Infection Spread*, 2) *Health Vulnerability*, 3) *Pop Health Risk*, and 4) *Mortality*.

As theoretically expected, *Infection Spread* was identified with large loadings by new cases (.90), new deaths (.92), new deaths per million (.81) and the stringency index (.56). *Mortality* was identified by total cases per million, total deaths per million and the stringency index. *Infection Spread* and *Mortality* were inter-correlated as expected, r = 0.30 (p < .01). *Health Vulnerability* was identified by percent of population aged 65 years or older, median age of the population and days since lockdown (negative path coefficient = -.76). *Pop Health Risk* was identified by cardiovascular death rate, ICU hospital beds per 100K, and days since lockdown. The two latent variables *Health Vulnerability* and *Pop Health Risk* were inter-correlated, r = .15 (p < .05). Overall, the latent variable path model fit the data well with the expected theoretical path coefficients and inter-correlations among the latent variables.

## Discussion

The major results are (1) a LVPA theoretical model of the covid-19 pandemic, and (2) a determination of the influence of the lockdown measures. The date of the lockdown for the mortality data for eight countries was minor but the stringency of the lockdown had a major impact. Similar results were obtained by Maier and Brockmann [21] in covid-19 data from China showing non-exponential spread that occurs when the supply of susceptible individuals is depleted on a time scale comparable to the infectious period of the virus.

### Logistic growth

The logistic growth data for the eight European countries in the present study revealed that the earliest lockdown was Italy (March 9) and the latest was the United Kingdom (March 24). Both the UK and Belgium had among the lowest stringency indices (33.56 and 35.84 respectively) thus accounting for the rapid exponential growth of deaths per million and new deaths per million due to poor containment [11, 12, 21].

### Stepwise multiple regressions

The most important effect was from the stringency index. Based on the regression equations, there was relatively high predictive efficiency for total deaths per million, total cases per million, total deaths, and new cases per million.

Besides the stringency index, other important variables that had an impact on outcomes were ICU hospital beds per100k, diabetes prevalence, gdp per capita, cardiovascular death rate, percentage of the population aged 65 or older, median age of the population, which are all suspected to have an impact on infection and mortality rates for covid-19 infections [22–24]. A recent British study, found that increasing age, male sex, population density, more deprived areas, and black ethnicity were associated with an increased risk of a positive SARS-CoV-2 test as well as chronic kidney disease and obesity [25].

### LVPA and a theoretical model

A theoretical model was tested in a confirmatory LVPA analysis (Fig 3); the four latent variables were confirmed. Both of the lockdown variables (days since lockdown and stringency index) behaved as theoretically expected s did the stringency index loading on both Infection Spread and Mortality The days since lock down loaded on *Health Vulnerability*.

The *Health Vulnerability* latent variable was identified by an aging population. There is also the large negative (-0.76; an inverse relationship) path coefficient to days since lockdown, as is theoretically expected. The *Pop Health Risk* latent variable was identified by the cardiovascular death rate and the available ICU hospital beds per 100K, and a medium path coefficient from days since lockdown (0.41), all theoretically coherent for this latent variable. *Health Vulnerability* and *Pop Health Risk* have a small but significant inter-correlation (r = 0.15, p < .05). The overall LVPA model is theoretically meaningful and coherent with acceptable fit indices and root mean square residual.

### Strengths and limitations

The present study has several strengths: (1) data from several countries, (2) precise indicators of containment, (3) multivariate statistical approach to the data, and (4) the development and fitting of a LVPA theoretical model of the covid-19 pandemic.

Limitations include: (1) these eight European countries are somewhat homogeneous compared to the variability of the rest of the world, (2) the lockdown dates were also quite similar varying only by days or weeks at most, and (3) other high risk health variables were not included (e.g., kidney disease, obesity).

## Conclusions

The results converged to indicate there was a larger effect from the stringency index on the growth of covid-19 infection and mortality rates. Notwithstanding the limitations of the relatively homogeneous European countries, it is evident that lockdown particularly the stringency, has a large impact on the spread of covid-19 virus and its mortality rate. Future research should incorporate data from other countries (e.g. USA, Brazil, Hong Kong, etc.) with much more variable lockdown dates and stringency indices.

## Supporting information

S1 Appendix Stringency index (OXBSG). (DOCX)

S2 Appendix Data columns and variables. (DOCX)

S3 Appendix Regression equations of the form Ŷ = β_1_X_1_ + β_2_X_2 …_ + β_K_X_K_ + C for covid-19 cases and deaths. (DOCX)

S4 Appendix Predictive efficiency of regression equations. (DOCX)

S5 Appendix Principal component structure matrix rotated to the oblimin criterion with Kaiser normalization. (DOCX)

S6 Appendix Lavaan R code for latent variable path analysis of covid-19 impact using Maximum Likelihood (ML) estimation. (DOCX)
