# Supplementary material for: Impact of the stringency of lockdown measures on covid-19: A theoretical model of a pandemic
Source: PLoS One. 2021 Oct 5;16(10):e0258205. doi: 10.1371/journal.pone.0258205 (PMC8491873; doi:10.1371/journal.pone.0258205)
Supplement: S1 Appendix — (DOCX) [file pone.0258205.s001.docx]

**Appendix A:**

<https://ourworldindata.org/grapher/covid-stringency-index?tab=chart>

STRINGENCY INDEX (OXBSG)

| Variable time span | Jan 1, 2020 – Jun 15, 2020 |
| --- | --- |
| Data published by | Thomas Hale, Sam Webster, Anna Petherick, Toby Phillips, and Beatriz Kira (2020). Oxford COVID-19 Government Response Tracker, Blavatnik School of Government. |
| Link | <https://www.bsg.ox.ac.uk/research/research-projects/oxford-covid-19-government-response-tracker> |

OxCGRT collects publicly available information on indicators of government response. These indicators take policies such as school closures, travel bans, etc. and record them on an ordinal scale; the remainder are financial indicators such as fiscal or monetary measures.

OxCGRT measures the variation in governments’ responses using its 'COVID-19 Government Response Stringency Index (Stringency Index)'. This composite measure is a simple additive score of nine indicators measured on an ordinal scale, rescaled to vary from 0 to 100. Please note that this measure is for comparative purposes only, and should not necessarily be interpreted as a rating of the appropriateness or effectiveness of a country's response.

Note:
This is an ongoing collation project of live data. If you see any inaccuracies in the underlying data please contact us on the feedback form below. The underlying index is evolving as the situation and data evolves from country to country, and will change over time as the data gets more accurate.

The specific policy and response categories are coded as follows:

School closures:
0 - No measures
1 - recommend closing
2 - Require closing (only some levels or categories,
eg just high school, or just public schools)
3 - Require closing all levels
No data - blank

Workplace closures:
0 - No measures
1 - recommend closing (or work from home)
2 - require closing (or work from home) for some
sectors or categories of workers
3 - require closing (or work from home) all but essential workplaces (eg grocery stores, doctors)
No data - blank

Cancel public events:
0- No measures
1 - Recommend cancelling
2 - Require cancelling
No data - blank

Restrictions on gatherings:
0 - No restrictions
1 - Restrictions on very large gatherings (the limit is above 1000 people)
2 - Restrictions on gatherings between 100-1000 people
3 - Restrictions on gatherings between 10-100 people
4 - Restrictions on gatherings of less than 10 people
No data - blank

Close public transport:
0 - No measures
1 - Recommend closing (or significantly reduce volume/route/means of transport available)
2 - Require closing (or prohibit most citizens from using it)

Public information campaigns:
0 -No COVID-19 public information campaign
1 - public officials urging caution about COVID-19
2 - coordinated public information campaign (e.g. across traditional and social media)
No data - blank

Stay at home:
0 - No measures
1 - recommend not leaving house
2 - require not leaving house with exceptions for daily exercise, grocery shopping, and ‘essential’ trips
3 - Require not leaving house with minimal exceptions (e.g. allowed to leave only once every few days, or only one person can leave at a time, etc.)
No data - blank

Restrictions on internal movement:
0 - No measures
1 - Recommend movement restriction
2 - Restrict movement

International travel controls:
0 - No measures
1 - Screening
2 - Quarantine arrivals from high-risk regions
3 - Ban on high-risk regions
4 - Total border closure
No data - blank

Testing policy
0 – No testing policy
1 – Only those who both (a) have symptoms AND (b) meet specific criteria (eg key workers, admitted to hospital, came into contact with a known case, returned from overseas)
2 – testing of anyone showing COVID-19 symptoms
3 – open public testing (eg “drive through” testing available to asymptomatic people)
No data

Contract tracing
0 - No contact tracing
1 - Limited contact tracing - not done for all cases
2 - Comprehensive contact tracing - done for all cases
No data
