# Supplementary material for: Impact of the stringency of lockdown measures on covid-19: A theoretical model of a pandemic
Source: PLoS One. 2021 Oct 5;16(10):e0258205. doi: 10.1371/journal.pone.0258205 (PMC8491873; doi:10.1371/journal.pone.0258205)
Supplement: S2 Appendix — (DOCX) [file pone.0258205.s002.docx]

**Appendix B:**

Data Columns and Variables

For the eight European countries (Austria, Belgium, France, Germany, Italy, Netherlands, Spain and United Kingdom) the data columns and variables are: iso_code, location, date, total_cases, new_cases, total_deaths, new_deaths, total_cases_per_million, new_cases_per_million, total_deaths_per_million, new_deaths_per_million, total_tests, new_tests, new_tests_smoothed, total_tests_per_thousand, new_tests_per_thousand, new_tests_smoothed_per_thousand, tests_units, stringency_index, population, population_density, median_age, aged_65_older, aged_70_older, gdp_per_capita, extreme_poverty, cvd_death_rate, diabetes_prevalence, female_smokers, male_smokers, handwashing_facilities, hospital_beds_per_100k

The codebook for these variables can be downloaded from: <https://github.com/owid/covid-19-data/blob/master/public/data/owid-covid-data-codebook.md> (accessed 06/01/2020)
