# Supplementary material for: Impact of the stringency of lockdown measures on covid-19: A theoretical model of a pandemic
Source: PLoS One. 2021 Oct 5;16(10):e0258205. doi: 10.1371/journal.pone.0258205 (PMC8491873; doi:10.1371/journal.pone.0258205)
Supplement: S3 Appendix — (DOCX) [file pone.0258205.s003.docx]

**Appendix C:**

Regression Equations of the form Ŷ = β_1_X_1_ + β_2_X_2 …_ + β_K_X_K_ + C for covid-19 cases and deaths

| **Part A: total deaths per million** |
| --- |
| **Regression Equation:** Ŷ (total deaths per million) = 3.43 x stringency index – 11.18 x ICU hospital beds per100k –17.90 x diabetes prevalence– 0.005 x gdp per capita + 343.06 |
| **Part B: total cases per million** |
| **Regression Equation:** Ŷ (total cases per million) = 28.98 x stringency index –91.27 x ICU hospital beds per100k + 34.00 x days since lockdown– 237.86 x aged 65 or older + 127.75 x median age – 3111.25 |
| **Part C: total deaths** |
| **Regression Equation:** Ŷ (total deaths) = 143.86 x stringency index- .534 x gdp per capita – 837.42 x ICU hospital beds per100k + 473.04 x aged 65 or older + 16226.13 |
| **Part D: new cases per million** |
| **Regression Equation:** Ŷ (new cases per million) = .596 x stringency index – 2.25 x ICU hospital beds per100k + .234 x CVD death rate - 2.83 x aged 65 or older - 0.001 x gdp per capita +66.89 |
