# Supplementary figures and images for: Impact of the stringency of lockdown measures on covid-19: A theoretical model of a pandemic
Source: PLoS One. 2021 Oct 5;16(10):e0258205. doi: 10.1371/journal.pone.0258205 (PMC8491873; doi:10.1371/journal.pone.0258205)

**Appendix D:**

Predictive efficiency of regression equations


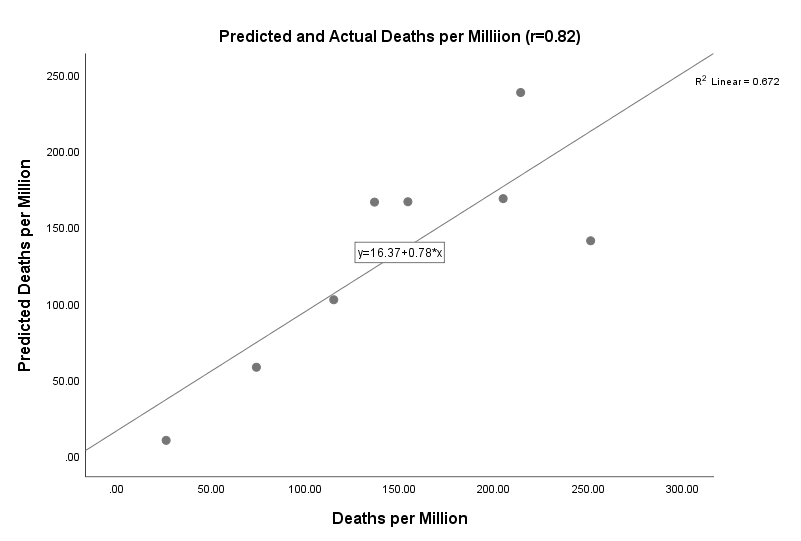


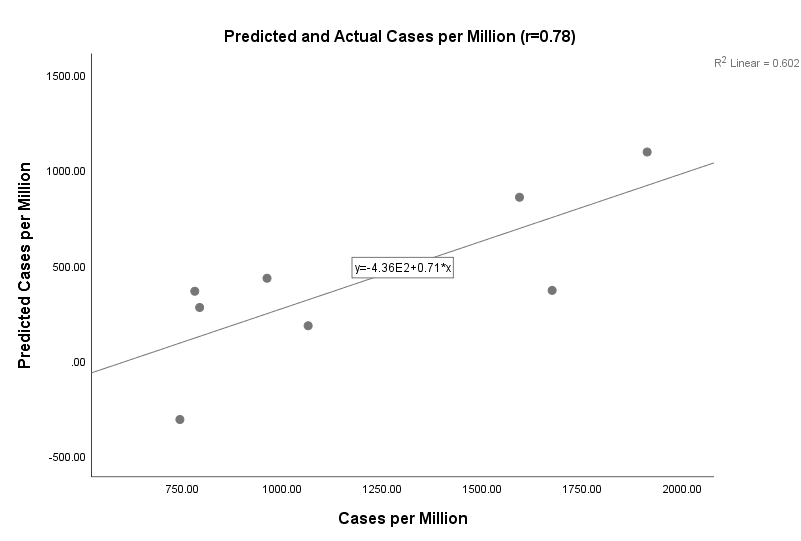


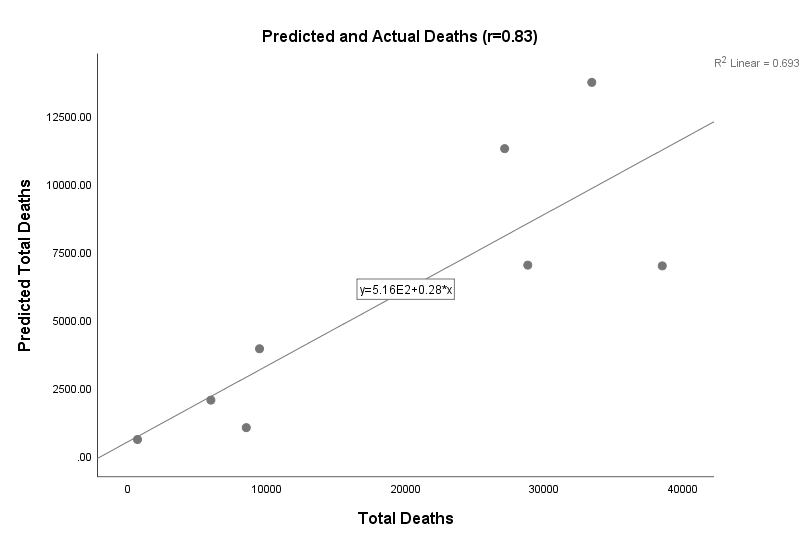


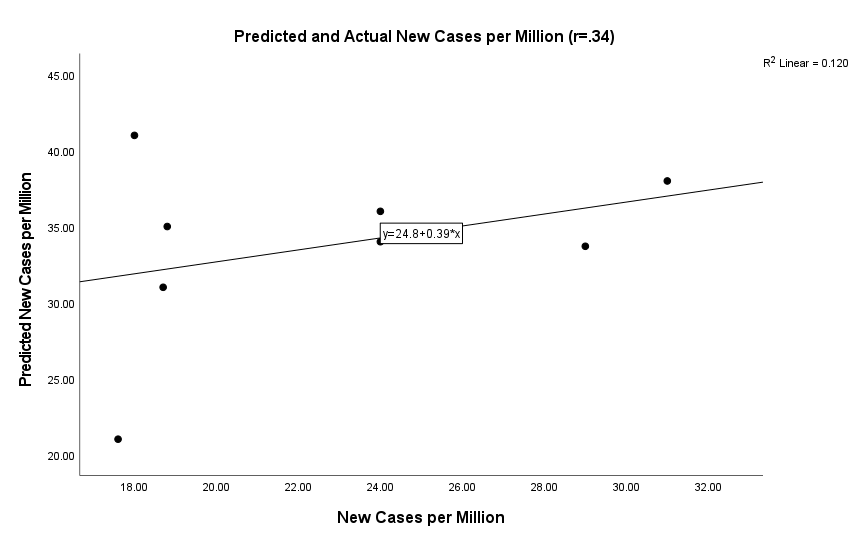

Supplement: S4 Appendix — (DOCX) [file pone.0258205.s004.docx]
