# Supplementary material for: Impact of the stringency of lockdown measures on covid-19: A theoretical model of a pandemic
Source: PLoS One. 2021 Oct 5;16(10):e0258205. doi: 10.1371/journal.pone.0258205 (PMC8491873; doi:10.1371/journal.pone.0258205)
Supplement: S5 Appendix — (DOCX) [file pone.0258205.s005.docx]

**Appendix E:**

Principal Component Structure Matrix Rotated to the Oblimin Criterion with Kaiser Normalization

| Variable | Component | | | |
| --- | --- | --- | --- | --- |
|  | Infection Spread | Health Vulnerability | Pop Health Risk | Mortality |
| Total deaths per million | .301 | .031 | -.262 | **.962** |
| Total cases per million | .359 | .090 | -.135 | **.981** |
| Stringency index | **.664** | .072 | .000 | **.768** |
| Days lockdown | .009 | **.757** | **-.402** | .209 |
| New deaths | **.938** | .032 | -.219 | .360 |
| New cases | **.903** | .097 | .014 | .267 |
| New deaths per million | **.861** | -.069 | -.228 | .201 |
| Aged 65 or older | .097 | **.927** | .166 | .008 |
| Median age | -.001 | **.930** | .261 | .007 |
| ICU hospital beds per 100k | -.171 | .039 | **.817** | -.161 |
| CV death rate | -.093 | .123 | **.914** | -.194 |
