# Supplementary material for: Impact of the stringency of lockdown measures on covid-19: A theoretical model of a pandemic
Source: PLoS One. 2021 Oct 5;16(10):e0258205. doi: 10.1371/journal.pone.0258205 (PMC8491873; doi:10.1371/journal.pone.0258205)
Supplement: S6 Appendix — (DOCX) [file pone.0258205.s006.docx]

**Appendix F:** lavaan R code for Latent Variable Path Analysis of covid-19 Impact Using Maximum Likelihood (ML) Estimation

R Code

> #### LVPA ####

> #### standardize the variables into z-scores ####

cvd <- read.csv("covid19.csv", header = T)

> names(cvd)

> cvdmodel2 <- '

+ f1 =~ total_deaths_per_million + total_cases_per_million + stringency_index + days_lck

+ f2 =~ new_deaths + stringency_index + new_cases + new_deaths_per_million

+ f3 =~ days_lck + aged_65_older + median_age

+ f4 =~ hospital_beds_per_100k + cvd_death_rate + median_age + aged_65_older

+ f1 ~~ f2

+ f3 ~~ f4'

> fit2 <- sem(cvdmodel2, data = cvd, se = "robust.sem", test = "standard", mimic = "EQS")

> summary(fit2, fit.measures = TRUE)

Model Fit Results

lavaan 0.6-6 ended normally after 55 iterations

Estimator ML

Optimization method NLMINB

Number of free parameters 32

Used Total

Number of observations 601 615

Model Test User Model:

Test statistic (Chi-square) 548.856

Degrees of freedom 34

P-value (Chi-square) 0.000

Model Test Baseline Model:

Test statistic 5435.273

Degrees of freedom 55

P-value 0.000

User Model versus Baseline Model:

Comparative Fit Index (CFI) 0.904

Tucker-Lewis Index (TLI) 0.845

Loglikelihood and Information Criteria:

Loglikelihood user model (H0) -6939.803

Loglikelihood unrestricted model (H1) -6664.917

Akaike (AIC) 13943.605

Bayesian (BIC) 14084.360

Sample-size adjusted Bayesian (BIC) 13982.769

Root Mean Square Error of Approximation:

RMSEA 0.159

90 Percent confidence interval - lower 0.147

90 Percent confidence interval - upper 0.171

P-value RMSEA <= 0.05 0.000

Standardized Root Mean Square Residual:

SRMR 0.083

Parameter Estimates:

Standard errors Robust.sem

Information Expected

Information saturated (h1) model Structured

Latent Variables:

Estimate Std.Err z-value P(>|z|)

f1 =~

ttl_dths_pr_ml 1.000

ttl_css_pr_mll 1.220 0.027 45.326 0.000

stringency_ndx 0.559 0.044 12.784 0.000

days_lck -0.002 0.023 -0.082 0.935

f2 =~

new_deaths 1.000

stringency_ndx 0.423 0.057 7.402 0.000

new_cases 0.849 0.090 9.412 0.000

nw_dths_pr_mll 0.835 0.031 26.828 0.000

f3 =~

days_lck 1.000

aged_65_older -1.243 0.038 -32.928 0.000

median_age -1.410 0.068 -20.831 0.000

f4 =~

hsptl_bds__100 1.000

cvd_death_rate 2.217 0.254 8.735 0.000

median_age 1.286 0.107 11.979 0.000

aged_65_older 1.010 0.084 12.093 0.000

Covariances:

Estimate Std.Err z-value P(>|z|)

f1 ~~

f2 0.288 0.041 6.972 0.000

f3 ~~

f4 0.147 0.027 5.382 0.000

f1 ~~

f3 -0.074 0.023 -3.239 0.001

f4 0.013 0.012 1.026 0.305

f2 ~~

f3 -0.061 0.036 -1.697 0.090

f4 -0.063 0.018 -3.577 0.000

Variances:

Estimate Std.Err z-value P(>|z|)

.ttl_dths_pr_ml 0.209 0.019 10.775 0.000

.ttl_css_pr_mll -0.139 0.017 -8.059 0.000

.stringency_ndx 0.455 0.026 17.221 0.000

.days_lck 0.462 0.021 22.310 0.000

.new_deaths 0.047 0.020 2.347 0.019

.new_cases 0.319 0.072 4.457 0.000

.nw_dths_pr_mll 0.344 0.093 3.691 0.000

.aged_65_older 0.273 0.015 18.163 0.000

.median_age 0.017 0.018 0.976 0.329

.hsptl_bds__100 0.743 0.027 27.897 0.000

.cvd_death_rate -0.286 0.093 -3.081 0.002

f1 0.752 0.066 11.428 0.000

f2 0.971 0.139 6.970 0.000

f3 0.546 0.058 9.385 0.000

f4 0.261 0.047 5.517 0.000
